# Milk Kept by Chloroform

**Published:** 1876-02

**Authors:** 


Milk Kept by Chloroform.—That milk can be kept sweet by the addition of a little chloroform, is a suggestion for which we have to thank J. P. Barnes, of London. When added in sufficient quantity to fresh milk, the lactic fermentation is prevented. To two-eight fluid ounces of fresh milk were added, respectively, ten and twenty minims of chloroform; they were kept in a warm place and constantly agitated. After five days had elapsed, that containing ten minims had developed lactic acid in quantity sufficient to separate the cæsein, while that containing twenty remained fresh and good. It might be found convenient to preserve milk in this manner; always taking care to boil it just before using, in order to drive off the chloroform.—*Ex.*

